# Cytomegalovirus and pregnancy: current evidence for clinical practice

**DOI:** 10.1590/1806-9282.20240509

**Published:** 2024-09-02

**Affiliations:** 1 Universidade Federal de São Paulo, Paulista School of Medicine, Department of Obstetrics – São Paulo (SP), Brazil.; 2 Ipiranga Hospital, Service of Gynecology and Obstetrics – São Paulo (SP), Brazil.; 3 Universidade Municipal de São Caetano do Sul, Discipline of Woman Health – São Caetano do Sul (SP), Brazil.

Cytomegalovirus (CMV) is an enveloped DNA virus that, due to several intrinsic characteristics, establishes itself in granulocytes and monocytes after primary infection and becomes a lifelong latent infection^1,2^. CMV is the most common congenital viral infection in the world, with a prevalence rate of approximately 0.5–2.0% among all live births^1-3^. CMV is the first cause of permanent sequelae in childhood, accounting for one-fourth of cases of congenital sensorineural hearing loss, 10% of cases of cerebral palsy, and severe neurological abnormalities, vision loss, and growth disorders^1-3^.

Global serum prevalence in women of childbearing age is approximately 86%^4^. This is important because only 50% of congenital CMV cases are maternal primary infections^5^. A Brazilian study confirmed the fact that most newborns affected by CMV come from previously infected/immune mothers (1–3% vertical transmission), but, in maternal primary infection, the vertical transmission rate is five times higher (30–40% vertical transmission)^6^.

CMV is transmitted by direct contact of mucous membranes with contaminated body fluids such as urine, saliva, blood, genital secretions, tears, contaminated breast milk, solid organ transplants, and stem cells^7-9^. Symptoms in immunocompetent individuals are few and nonspecific or absent, but it can cause severe disease in immunosuppressed individuals, including fetuses^1,8^. There is no vaccine for CMV, despite numerous ongoing studies^10^. Until 2020, it was believed that the only way to prevent vertical transmission of CMV was through behavioral measures such as hand hygiene, avoiding contact with children's diapers, and avoiding kissing young children^10^.

Until 2022, no guideline published in English suggested testing for CMV in prenatal care^7^. Reasons varied, including lack of vaccine, difficulty interpreting tests, inability to treat, and lack of randomized controlled trials^7^. Eventually, serologies were requested by physicians at random or when CMV was suspected because of maternal symptoms, contact with children with symptoms, or fetal findings suggestive of CMV^7^.

The research on CMV in pregnancy is carried out mainly through specific antibody tests (IgG, IgM, and IgG avidity) or by detecting CMV DNA in body fluids (blood, urine, and saliva)^3^. Table 1 summarizes maternal serologies and how to interpret the results. Congenital CMV infection can damage the fetus directly or indirectly through placental dysfunction, resulting in miscarriage, preterm birth, or fetal growth restriction (FGR)^10,11^. The gestational age can influence vertical transmission, being higher with the progression of pregnancy^10,12^. When the virus crosses the placental barrier, the first fetal organ to be infected replicates in the tubular epithelium of the fetal kidney, with tropism for reticuloendothelial cells and the central nervous system (CNS)^10,12^. Shahar-Nissan et al.^13^ describe that there is a cascade of events that culminate in fetal infection. This cascade of events can take 7–8 weeks, and it is described as maternal viremia, placental infection, and fetal dissemination via the hematogenous route. Therefore, amniotic fluid testing should be performed 8 weeks after the presumed period of infection and preferably after 22 weeks of gestation to reduce the risk of false negative results^10,14^. In newborns, it is performed by viral detection in body fluids (urine, saliva, and blood) by PCR, culture, or antigen testing until 3 weeks of life^2,10^. After this period, it is difficult to distinguish congenital from acquired postnatal infection^2,10^.

**Table 1:** Maternal serology and interpretation of the results.

Serology	Interpretation of the results
IgG - IgM -	Susceptible
IgG + IgM -	Immune/previous contact
IgG + IgM + high IgG avidity	Infection older than 12 weeks
IgG + IgM + low IgG avidity	Infection less than 12 weeks old
IgG - IgM + IgG + IgM + after 15 days IgG - IgM + after 15 days	Possible recent infection, repeat serology in 15 days Recent infection/seroconversion False positive for cytomegalovirus

Chatzakis et al.^12^ in a meta-analysis, divided the fetal findings according to the period of maternal infection: periconceptional (4 weeks before to 3–6 weeks after the last menstrual period), first (6–13 weeks), second (14–26 weeks), and third trimester (>26 weeks). Fetal abnormalities were limited to periconceptional and first-trimester infections with rates of 28.8, 19.3, 0.9, and 0.4% for periconceptional, first-, second-, and third-trimester infections, respectively.

When the virus crosses the placental barrier and reaches the fetus, fetal damage is progressive and the first ultrasound findings are usually due to systemic infection and nonspecific (FGR, abnormal amniotic fluid volume, ascites, pleural effusion, skin edema, hydrops, placentomegaly, hyperechogenic bowel, splenomegaly, liver calcifications)^15^. CNS findings usually occur after weeks, and severe brain involvement is usually a predictor of poor prognosis, with microcephaly being the only finding that actually predicts an unfavorable outcome in up to 95% of cases^15,16^. The most common ultrasound findings are ventriculomegaly, periventricular changes, temporal cysts, and brain parenchymal lesions^16^.

Since 2005, when Nigro et al.^17^ published a nonrandomized study proposing the use of hyperimmune globulin for the treatment and prevention of congenital CMV, several promising studies have been published. The efficacy of hyperimmune globulin has not been proven in subsequent studies^18,19^; however, high-dose valacyclovir has been shown in several studies and systematic reviews to be effective and safe in preventing vertical transmission of CMV in primary maternal infections acquired during the periconceptional period and the first trimester of pregnancy^3,5,13,15,20-24^. Acyclovir is the precursor drug to valacyclovir and is converted to acyclovir in the first hepatic passage. Valacyclovir has been a drug of choice for herpes virus infections as it is more effective than acyclovir^24^, which is classified as class B in pregnancy^22^. Treatment with valacyclovir is contraindicated in people who are unable to swallow capsules, in cases of severe vomiting, pre-existing liver disease, renal dysfunction, bone marrow suppression, patients receiving immunotherapy, or in cases of hypersensitivity to acyclovir^13^. The most common adverse reactions of valacyclovir are thrombocytopenia (usually mild), nausea, headache, abdominal pain, and nonspecific rash, none of which were significant and did not require discontinuation of the drug in a study by Shahar-Nissan et al.^13^.

In 2016, Leruez-Ville et al.^15^ in their nonrandomized study, showed a reduction in asymptomatic newborns from 43% (no treatment) to 82% with the use of high-dose valacyclovir (8 g/day) in fetuses with extra-brain and brain findings suggestive of vertical transmission of CMV. In 2020, Shahar-Nissan et al.^13^ published a double-blind, randomized trial of valacyclovir (8 g/day) for the prevention of CMV congenital infection acquired periconceptionally or in the first trimester. The amniotic fluid PCR positivity rate was 30% in the control group compared with 11% in the treated group. Since this publication, at least six large studies, including meta-analyses and phase 3 trials, have been published confirming the use of valacyclovir 8 g/day for the prevention of CMV vertical transmission of maternal primary infection in the early stages of pregnancy (periconceptional and first trimester)^3,5,15,20-24^.

**Figure 1:**
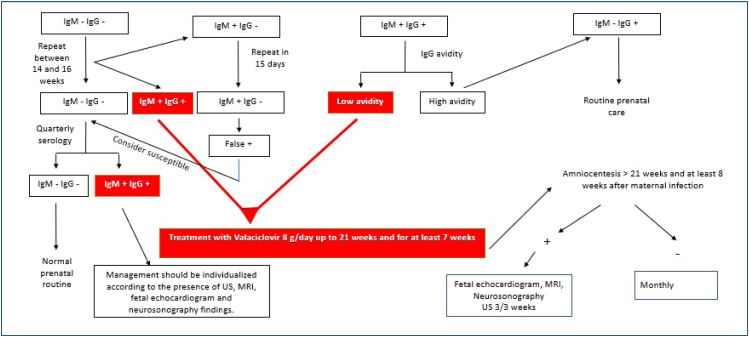
Flowchart of the suggested follow-up and treatment according to the maternal serologies during prenatal care.

Given the serious consequences of congenital CMV, the number of children worldwide who develop permanent and often severe sequelae each year, and the high prevalence of CMV in the population together with the strong evidence that valacyclovir is effective and safe in preventing vertical transmission, we suggest that protocols be revised to include routine CMV serology in the prenatal period (first visit and repeated at 12–14 weeks) when resources are available and especially in the event of seroconversion. Treatment with valacyclovir 8 g/day (4 g 12/12 h) should be started for at least 7 weeks after the estimated date of seroconversion and until at least 21 weeks when amniocentesis for amniotic fluid PCR for CMV is indicated^3,5,13,15,20-24^. Figure 1 shows suggested follow-up and treatment according to the serologies found during prenatal care.
